# Factors associated with HPV vaccine acceptability and hesitancy among Black mothers with young daughters in the United States

**DOI:** 10.3389/fpubh.2023.1124206

**Published:** 2023-04-17

**Authors:** Aaliyah Gray, Celia B. Fisher

**Affiliations:** ^1^Department of Psychology, Fordham University, Bronx, NY, United States; ^2^Department of Epidemiology, Florida International University, Miami, FL, United States; ^3^Center for Ethics Education, Fordham University, Bronx, NY, United States

**Keywords:** HPV, Black, African American, mothers, pediatric, vaccine hesitancy, vaccination intentions, attitudes

## Abstract

**Introduction:**

Compared to other-race peers, Black women are disproportionately impacted by human papillomavirus [HPV] infection, related health outcomes, and cervical cancer mortality as a result of suboptimal HPV vaccine uptake during adolescence. Few studies in the United States have examined psychosocial determinants of HPV vaccine acceptability and hesitancy among Black parents. The current study integrated the health belief model and the theory of planned behavior to evaluate the extent to which psychosocial factors are associated with pediatric HPV vaccination intentions among this population.

**Methods:**

Black mothers (*N* = 402; age range = 25 to 69 years, *M* = 37.45, *SD* = 7.88) of daughters ages 9 to 15 years completed an online survey assessing HPV infection and vaccine beliefs and attitudes across four domains: Mother’s HPV Perceptions, Mother’s Vaccine Attitudes, Cues to Action, and Perceived Barriers to HPV Vaccination. Participants indicated their willingness to vaccinate their daughter on a 5-level ordinal scale (“I will definitely not have my daughter get the vaccine” to “I will definitely have my daughter get the vaccine”) which was dichotomously recoded for binomial logistic regressions.

**Results:**

Half of the sample (48%) intended to vaccinate their daughter. Number of daughters, mother’s HPV vaccine status, perceived HPV vaccine benefits, HPV vaccine safety concerns, pediatric HPV vaccine peer norms, and doctor recommendations emerged as independent factors of Black mothers’ intentions to vaccinate their daughters against HPV when controlling for all other factors.

**Discussion:**

In addition to medical training to increase doctor recommendation of the HPV vaccine for Black girls, population-tailored public health messaging aimed at promoting HPV vaccine acceptance among Black mothers is urgently needed. This messaging should engage community support and emphasize the benefits of vaccination for adolescent Black girls while also addressing parental concerns regarding the safety of pediatric HPV vaccination.

## 1. Introduction

Human papillomavirus [HPV] infections are the most common sexually transmitted infections in the United States [U.S.] with some estimates indicating nearly all people will contract at least one type of sexually-transmitted HPV (1). Adolescents and young adults (i.e., 18–25 years) are at particular risk; more than 60% of pre-teen and teen girls are diagnosed with an HPV infection and research suggests 50–80% of people test positive for HPV within 2–3 years of the first time they engaged in sexual activity (2). Unfortunately, approximately half of the sexually-transmitted types of HPV infections among young female adolescents and young adults are responsible for a large percentage of cervical and genital cancers and cancer-related mortality among adult women worldwide (1, 3, 4). Since it was introduced in 2006, HPV vaccines have been found to have high efficacy for prevention of HPV infection, and therefore, cancers caused by HPV infection (5). A two-dose schedule is recommended for those who get the first dose before their 15th birthday (6). Despite incremental increases in HPV vaccination uptake among children and adolescents since 2006, HPV vaccination remains lower than other pediatric vaccines in the United States (7). Consequently, there is an urgent need to increase HPV vaccination rates among girls between the ages of 9 and 15 prior to sexual debut in order to prevent infection and HPV-related cancer as they grow older (8, 9).

Not all women face the same risk for HPV infection. In the United States, Black women are disproportionately impacted by the transmission of HPV, face greater risk of HPV-related outcomes ranging from genital warts to various cancers, and have the highest mortality rate of cervical cancer (10–12). Data also suggest that high-risk HPV infections take longer to clear for Black women compared to White women (13). Further, Black girls are more likely than White and Asian race peers to report early sexual debut, suggesting it is more likely for HPV exposure to occur earlier in development for Black girls (14, 15). Although early vaccination for young Black girls is paramount to prevention of HPV transmission, they are particularly vulnerable to HPV vaccination delay or not receiving the vaccine at all. Black girls are less likely to initiate or complete the recommended vaccination series than peers of other races and ethnicities (16–19). Compared to 80% of Black girls who initiated the vaccine series and 64% who were up-to-date in 2020, 84% of Hispanic and 91.8% of American Indian/Alaska Native girls initiated and 68 and 72%, respectively, were up-to-date in 2020 (20). During adolescence, parents are responsible for the decision to vaccinate their daughters against HPV. Across racial and ethnic groups, delay in HPV is associated with sociodemographic characteristics including parent education level, household income, or differential access to health care services (21). However, these factors do not fully explain HPV vaccination inequities among Black girls (21). As such, investigation of additional social determinants of HPV vaccine acceptability and hesitancy among Black parents is imperative (17, 22).

Vaccine hesitancy is the refusal or delay in the acceptance of a vaccination despite availability of the vaccine or vaccination services (23). Studies of parental HPV vaccine acceptability and hesitancy have drawn on the health belief model (24) and the theory of planned behavior (25) to explore psychosocial factors contributing to parental decision-making. When combined, constructs of these theories overlap to provide a holistic psychosocial perspective of factors that likely contribute to parental HPV vaccination acceptance. These factors include parental knowledge of HPV infection and the HPV vaccine, parents’ perception of their daughter’s susceptibility to HPV infection and severity of HPV infection to their daughter’s health (26–30). Acceptability among parents is also associated with perceived health benefits of receiving the HPV vaccine, positive attitudes toward pediatric vaccines in general, and perceived community support and favorable norms surrounding pediatric vaccination against HPV (26, 29–34). Parents who feel efficacious to request the vaccine and who perceive that their daughter’s doctor recommends and supports the HPV vaccine are also more likely to intend to vaccinate their child (27, 29, 33, 34). On the other hand, vaccine hesitancy has been associated with substantial structural and psychological barriers including perceived inaccessibility of the vaccine, concerns regarding the safety of the HPV vaccine, and concerns about sexual disinhibition and sexual stigma among daughters who receive the HPV vaccine (26–29, 31–33).

Black parents in the United States, however, have been severely underrepresented in research examining attitudes and beliefs regarding pediatric HPV vaccination. Most research in the United States has drawn on samples consisting of largely non-Hispanic white populations and has not examined potential racial/ethnic similarities or differences given small minority sample sizes. Consequently, little is known about the psychosocial factors underlying HPV vaccination intentions and related attitudes among Black parents. What is known about Black parents’ attitudes toward pediatric HPV vaccination primarily draws on a few qualitative studies. The themes reported in these studies suggest that Black parents are influenced by not only the constructs identified in the health belief and theory of planned behavior models, but also systemic barriers and sociocultural factors. For example, some Black parents report that although HPV-specific knowledge would be central to their decision-making process (35), they feel they lack access to adequate knowledge to make an informed decision (36). Others are concerned that the vaccine is too new to be safe, fear potential side effects will have long-term harmful impacts on their daughter’s reproductive health, and refer to an overall sense of cultural medical mistrust based on historical and contemporary medical abuses experienced by Black peoples in the United States (36–39). Still, others worry that giving their child the vaccine will reinforce social stereotypes regarding Black female promiscuity (38, 39). By contrast, parents who are more accepting of the vaccine report they are motivated by concerns that the HPV infections pose severe health consequences to their daughters (38, 40, 41), and that hearing about the vaccine at church, seeing other Black parents vaccinate their daughters, and receiving recommendations from trusted providers positively influence their acceptance (37, 39, 40). Given the lack of quantitative data on Black mothers with unvaccinated children in the United States, current interventions to promote HPV vaccination intentions among Black parents are likely to be uninformed by the unique issues and concerns that must be considered among this population of parents. Understanding what factors are associated with Black mother’s intentions to vaccinate their daughters is central to improving vaccine uptake among this population, and thus, reducing disparities in HPV transmission and outcomes for Black girls and women.

### 1.1. The current study

**Figure 1:**
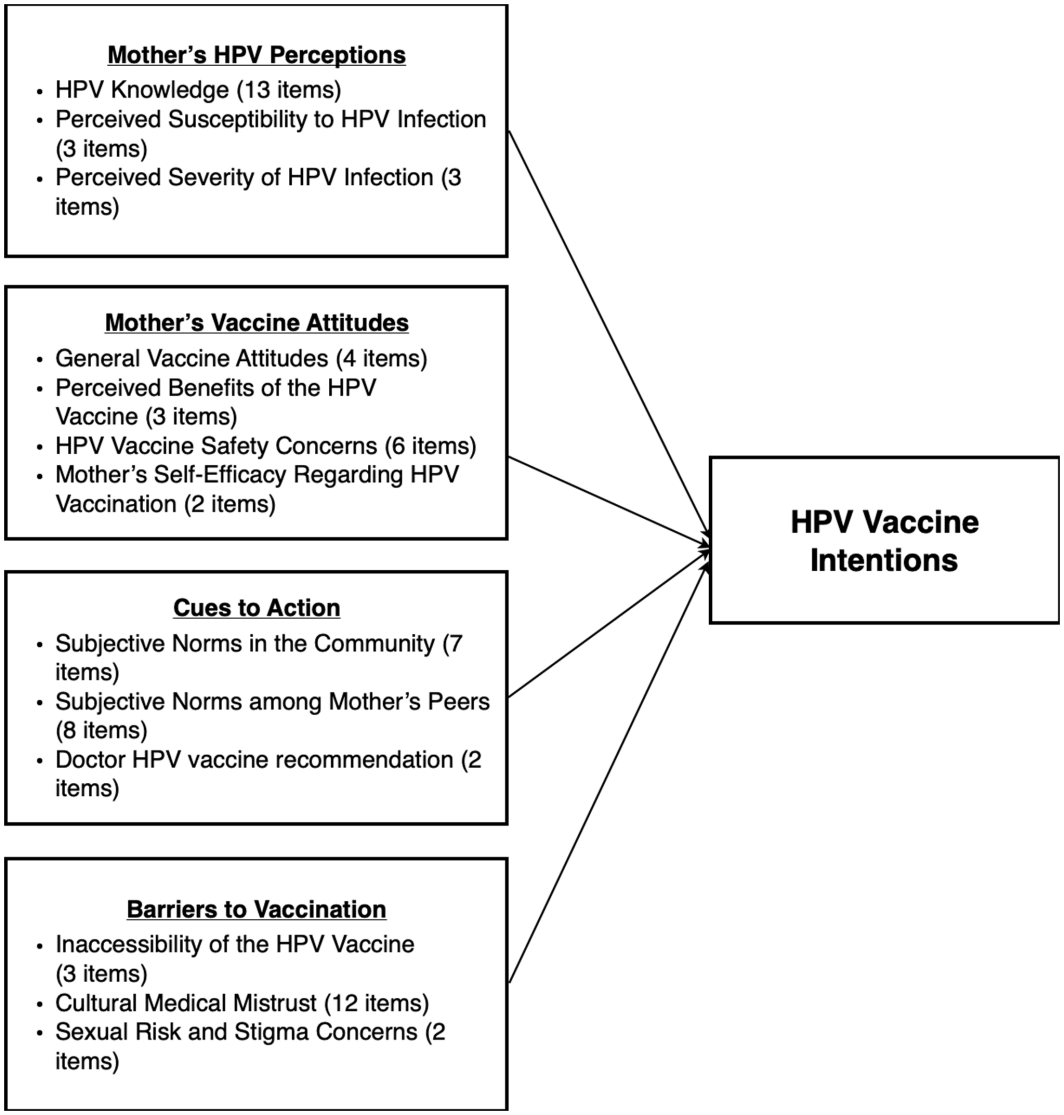
Integrated model of psychosocial determinants of HPV vaccination intentions among Black mothers. Culturally informed theoretical model integrating components of the health belief model and the theory of planned behavior as psychosocial determinants of HPV vaccine intentions among Black mothers.

A key goal of the 2020 Global Strategy to Accelerate the Elimination of Cervical Cancer is the complete vaccination of 90% of girls between ages 9 and 15 by the year 2030 (42). As of June 2020, half of WHO member states have introduced the HPV vaccine with a majority of these countries located in the Americas and Europe (85% and 77%, respectively) and the least in Africa (31%) (43). However, a substantial reduction in HPV vaccine coverage in the United States and globally has been a consequence of the COVID-19 pandemic (43, 44). In the United States, the 2020 NIS-Teen [National Immunization Survey-Teen] survey found that HPV vaccine initiation in 2020 was lower than rates observed in 2019 for adolescents ages 13–17 and one study estimated that HPV vaccination decreased by 24% from 2019–2020 among adolescents ages 9–16 (45, 46). Given this context, effort must be made to strengthen acceptability and improve uptake of the HPV vaccine among Black girls to meet this goal. Vaccine hesitancy among Black parents magnifies disparities in HPV infection and HPV-related outcomes disproportionally borne by Black girls and ultimately underscores the importance of investigating factors related to Black parents’ intentions to vaccinate their daughters against HPV. As such, the objective of the present study is to draw on constructs suggested by a culturally informed health belief model and theory of planned behavior (see Figure 1) to quantitatively examine the extent to which these factors facilitate or hinder HPV vaccination intentions among Black mothers.

## 2. Methods

### 2.1. Participants

The current study surveyed Black female or woman-identifying primary guardians (referred to as “mothers” in this article) of adolescent young girls who have not received the HPV vaccine. We focus on mothers because research suggests they are typically the primary parent responsible for health care decisions regarding their children and make nearly 80% of these health care decisions (39, 47). Inclusion criteria for the current sample include: (1) self-reported age of 25 years old or older, (2) primary identification as Black (African American, Caribbean, African), (3) at least one daughter between the ages of 9 and 15, (4) identification as the mother, grandmother, aunt, or other female/woman guardian, (5) current residence in the United States, and (6) English competency.

### 2.2. Study procedures

**Figure 2:**
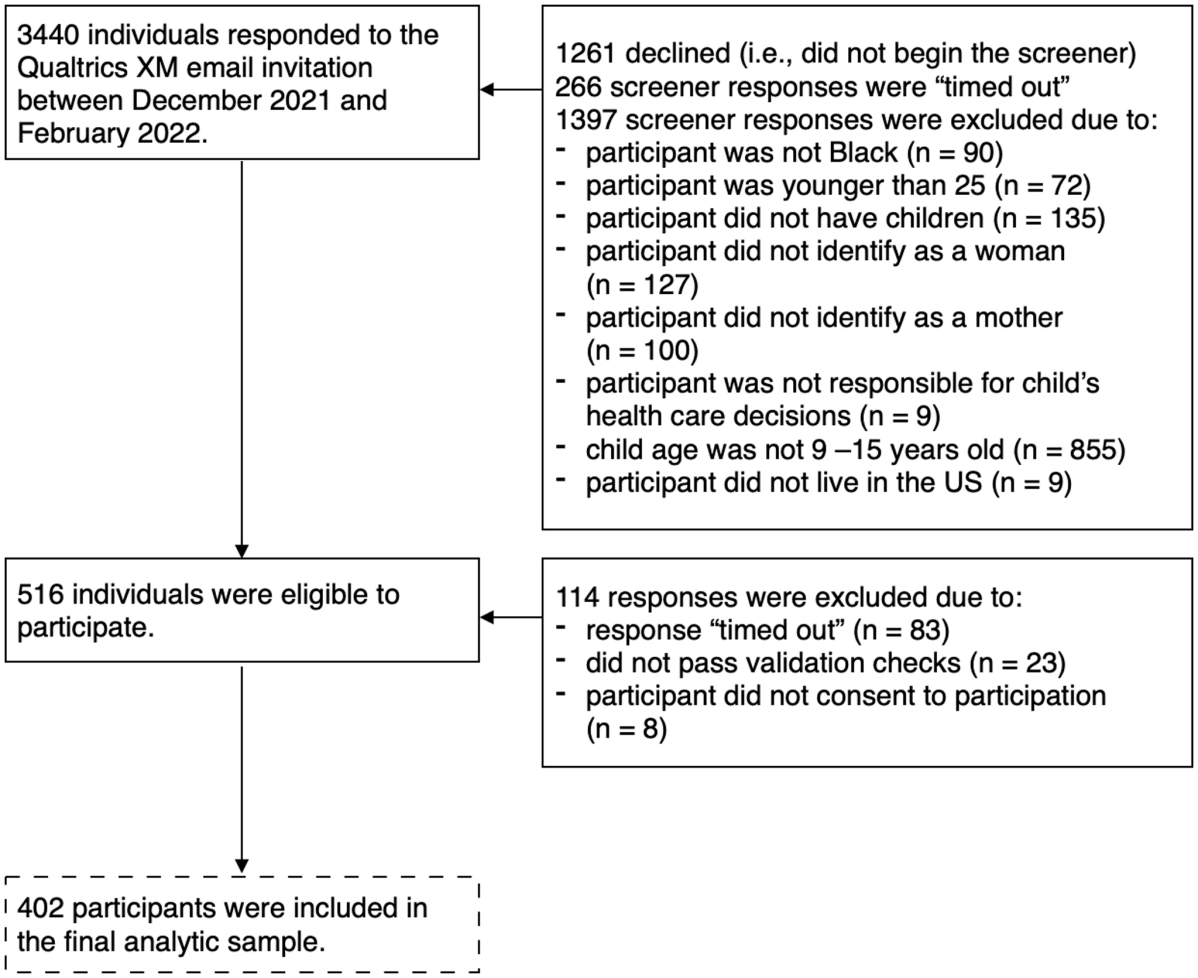
Flow chart of participation selection for the current study.

Data were collected between December 2021 and February 2022. Purposive recruitment of Black mothers across the United States was conducted by Qualtrics XM which sent emails to potential participants from survey panels of individuals interested in taking paid surveys. A 17-item screener determined eligibility based on age, gender, ethnicity/race, education level, parental role, age and gender of children, daughter’s HPV vaccine status (if applicable), and English competency. A total of 3,440 individuals responded to the Qualtrics XM email invitation and 516 (23.7%) individuals who began the screener were eligible based on inclusion criteria. The final sample included 402 participants who completed the full survey (see Figure 2 for full participant flow). Informed consent was provided to all participants who completed the screener and met inclusion criteria. The informed consent materials stated the purpose of the study, the role of participants in the study, potential risks and benefits of participation, confidentiality protections, and information regarding compensation. Participants indicated their consent by selecting “I agree” and proceeding to the full survey which comprised 92 items and took approximately 15–20 min complete (Median = 17.88 min). Participants who provided valid responses were compensated with a previously agreed-upon amount of points that could be exchanged for gift cards. Qualtrics performed various data integrity checks such as infrequency between survey responses and average of time for survey completion to determine validity of responses before issuing compensation to participants. There were no identifying links between any respondent’s Qualtrics screener or survey and their survey panel account. All study procedures and materials were approved by the Fordham University IRB.

### 2.3. Measures

#### 2.3.1. HPV vaccination intentions

The main outcome in the current study was measured as a single 5-level ordinal item assessing HPV vaccination intentions (27). For consistency in the wording of the response options, the current study modified the wording of the options to: “I will definitely not have my daughter get the vaccine,” “I will probably not have my daughter get the vaccine,” “I am thinking about getting my daughter the vaccine but I am unsure,” “I will probably have my daughter get the vaccine,” and “I will definitely have my daughter get the vaccine.” In the current study, participants who would probably or definitely vaccinate their daughter were categorized as “Intends to vaccinate” and those who would definitely not, probably not, or were unsure about the vaccine were categorized as “Does not intend to vaccinate.”

#### 2.3.2. Mother’s HPV perceptions

*HPV knowledge* was assessed with a 13-item true-false questionnaire (41). One item was updated for the current study to reflect current HPV recommendations for adult women (“The HPV vaccine is recommended for most adult women who are not sexually active or have not been vaccinated yet”). A score of 10 points (80%) or higher indicating a high level of knowledge. Inter-item reliability indicated items were moderately related, *ρ*_KR20_ = 0.56.

*Perceived susceptibility to HPV infection among daughters* was assessed with three items created for the current study (“I worry that my daughter will become infected with HPV once she is sexually active; I worry that my daughter will develop genital warts due to an HPV infection once she is sexually active; I worry that my daughter will develop HPV-related cancer in the future once she is sexually active”) based on items previously validated to examine perceived severity of HPV infection (48). A higher score indicated greater perceived susceptibility to HPV infection and was excellent (*α* = 0.91).

*Perceived severity of HPV infection among daughters* was assessed with three items (“An HPV infection could cause serious health problems for my daughter in the future; Genital warts caused by an HPV infection could cause serious health problems for my daughter in the future; HPV-related cancer could cause serious health problems for my daughter in the future”) (48). A higher score indicated greater perceived severity of HPV infection and reliability was excellent (*α* = 0.91).

#### 2.3.3. Mother’s vaccine attitudes

*General vaccination attitudes* assessed parental attitudes about pediatric vaccines with four items measuring positive (e.g., “I feel that vaccinating children is a good idea”) and negative attitudes (e.g., “I feel that doctors give out too many vaccinations”) (48). Negative items were reversed scored where a higher score indicates more positive vaccination attitudes. Reliability was acceptable (*α* = 0.79).

*Perceived benefits of the HPV vaccine* were assessed with three items to measure parents’ perceptions of how effective the HPV vaccine is for preventing infection, genital warts, and HPV-related cancer (“I feel that the HPV vaccination significantly reduces my daughter’s risk of HPV infection when she is older; I feel that the HPV vaccination significantly reduces my daughter’s risk of genital warts when she is older; I feel that the HPV vaccination significantly reduces my daughter’s risk of HPV-related cancer when she is older”) (48). A higher score indicated greater perceived benefit of the HPV vaccine and was excellent (*α* = 0.91).

*HPV vaccine safety concerns* were assessed using six items (e.g., “I feel that giving my daughter the HPV vaccine would be like performing an experiment on her”) (48) and an additional seventh item examining caregiver concerns regarding HPV vaccine side effects on fertility for daughters ages (“I feel that the HPV vaccine may cause problems getting pregnant in the future”) (31). A higher score indicated greater safety concerns and reliability for the 7 items was excellent (*α* = 0.94).

*Mother’s self-efficacy regarding HPV vaccination* was assessed with two items (“I am sure that I can request the HPV vaccine for my daughter even if her doctor does not bring it up; I am sure that I can ask my daughter’s doctor questions about the HPV vaccine”) (27). A higher score indicated greater perceived efficacy and reliability was good (*α* = 0.84).

#### 2.3.4. Cues to action

*Subjective norms in the community* were assessed with a single composite score comprised of 7 items measuring whether parents believed community members (e.g., religious leaders) support HPV vaccination for young girls (49). A higher score indicates greater perceived support for the HPV vaccination in the community and reliability was acceptable (*α* = 0.79).

*Subjective norms among mother’s peers* were assessed with seven of eight items measuring parents’ perceptions of HPV vaccine acceptability among peers (e.g., “Other parents in my community are getting their daughters the HPV vaccine”) (48). The additional item assessing co-parent support was not included as a part of the scale in the current study because it was likely that not all participants had a co-parent. A higher score indicated greater perceived support for the HPV vaccine among peers and reliability was good (*α* = 0.88).

*Doctor recommendation of HPV vaccine* was measured with two items. The first item was created for the current study (“In the past year, has your daughter’s doctor recommended the HPV vaccine to you?”) and provided three response options (“Has not mentioned or recommended the HPV vaccine,” “Has mentioned, but did not recommend the HPV vaccine,” and “Has mentioned and did recommend the HPV vaccine”). The second item assessed the perceived influence of doctor recommendations (“Thinking about your daughter’s doctor, how much will their opinion influence your decision about getting your daughter vaccinated against HPV?”) (26). In the current study, doctor recommendation and doctor influence were multiplied to create an interaction score assessing the influence of doctor recommendation of the HPV vaccine.

#### 2.3.5. Perceived barriers to HPV vaccination

*Inaccessibility of the HPV vaccine* was assessed with three items (“The cost of the HPV vaccine would keep me from having my daughter vaccinated,” “I do not know where to go for the HPV vaccine,” and “Transportation issues would prevent me from having my daughter vaccinated”) (41) and a fourth item assessing the burden posed to mothers by vaccination completion (“Having to take my daughter to the doctor two times six months apart or three time six months apart to get all required HPV vaccine shots would keep me from having my daughter vaccinated”) (27). A higher score indicated greater inaccessibility and reliability for the four items was acceptable (*α* = 0.76).

*Cultural medical mistrust* was assessed with the 12-item Group Based Medical Mistrust Scale (50) which includes negative (e.g., “Black people cannot trust doctors and healthcare workers”) and positive items (e.g., “Black people are treated the same as people of other groups by doctors and healthcare workers”). Positive items were reverse scored. A higher score indicated greater medical mistrust and reliability was excellent (*α* = 0.90).

*Sexual risk taking and sexual stigma concerns* were measured with two items (“I am concerned that if my daughter receives the HPV vaccine, she will think it is okay for her to have sex” and “I am concerned that if my daughter receives the HPV vaccine, she will think she does not have to use safe sex practices when she does become sexually active”) (26) and an additional third item addressing stigma adapted from the Sexual Self-Monitoring scale (“I am concerned that if my daughter receives the HPV vaccine, her pediatrician or healthcare provider will think she is sexually active”) (51). A higher score indicated greater sexual risk and stigma concerns and reliability for the three items was good (*α* = 0.86).

#### 2.3.6. Demographic variables and other participant characteristics

Mother-specific demographics and characteristics included self-reported age, highest level of obtained education, employment status, annual household income and subjective financial security, number of daughters ages 9–15 years, HPV infection and HPV vaccine awareness, vaccine history including HPV vaccination status, and marital status and co-parent support, if applicable. Understanding of HPV infection and awareness of the HPV vaccine were measured with two dichotomous (yes or no) items (27). Participant vaccine history was assessed with an inventory which included common routine and elective vaccines including tetanus, diphtheria, pertussis (whopping cough), seasonal flu, varicella (chicken pox), MMR (measles, mumps, and rubella), hepatitis A or B, pneumococcal (pneumonia and meningitis), polio, and rotavirus (49). Mothers also indicated whether they received the COVID-19 vaccine. Additionally, they indicated whether or not they had received the HPV vaccine. Lastly, they indicated whether or not they shared parenting responsibilities with a co-parent; those who reported a co-parent were asked whether they perceived co-parent support for vaccinating their daughter on a six-point scale ranging from [1] strongly disagree to [6] strongly agree. Four items assessed whether mothers personally experienced or were familiar with family or friend experiences with abnormal pap smears, genital warts, sexually transmitted infections [STIs], or cervical cancer or other HPV-related cancer diagnosis (30). Child-specific demographics included age, insurance status (“public,” “private,” or “uninsured”), pre-existing health conditions, and routine vaccination history which included tetanus, diphtheria, pertussis (whopping cough), seasonal flu, varicella (chicken pox), MMR (measles, mumps, and rubella), hepatitis A or B, pneumococcal (pneumonia and meningitis), polio, and rotavirus (49).

### 2.4. Data analysis

*A priori* G*Power analyses were conducted to determine the size of the sample needed to detect a significant effect with an alpha level of *p* = 0.05 and a power level of 1 − *β* = 0.80 for a two-sided binomial logistic regression where the suggested sample size was 324. The current sample of 402 mothers is sufficient. There was no missing data in the current study. All analyses were conducted in SPSS 27. Variables were described with frequencies and percentages or means and standard deviations as appropriate. Likewise, all continuous variables were screened for outliers and normal distribution. Exploratory independent t-tests and Chi-square tests of independence with adjusted standardized residuals were conducted to preliminarily examine (a) demographics and participant (i.e., mother-specific and daughter-specific) characteristics (Table 1) and (b) the hypothesized factors of HPV vaccine intentions (Table 2). A series of unadjusted logistic regressions were conducted to estimate the magnitude and direction of the associations between each independent factor and HPV vaccine intentions (Table 3). Lastly, a multivariable stepwise logistic regression was conducted to examine the extent to which the hypothesized factors estimate the odds of intending to vaccinate above and beyond other factors included in the model (Table 4). Mother-specific and daughter-specific covariates were entered as Step 1, Mother’s HPV Perceptions as Step 2, Mother’s Vaccine Attitudes as Step 3, Cues to Action as Step 4, and lastly, Perceived Barriers to Vaccination as Step 5. Nagelkerke’s pseudo *R*^2^ reported the overall explained variance of the model and the unique contribution of each step and Hosmer and Lemeshow tests assessed goodness-of-fit for each step.

**Table 1:** Differences in demographics and participant characteristics by HPV vaccine intentions.

	Total sample *N* = 402	Intends to vaccinate *N* = 193 (48%)	Does not intend to vaccinate *N* = 209 (52%)	*p* value
	*N* (%)	*N* (%)	*N* (%)	
*Mother’s age*	*M* = 37.45 (SD = 7.88)	*M* = 37.90 (SD = 7.70)	*M* = 37.03 (SD = 8.05)	0.27
*Education level*				0.62
8th grade or less	2 (0.5%)	1 (0.5%)	1 (0.5%)	
Partial high school	12 (3%)	6 (3.1%)	6 (2.9%)	
High school graduate	106 (26.4%)	49 (25.4%)	57 (27.3%)	
Partial college (at least 1 year)	142 (35.3%)	62 (32.1%)	80 (38.3%)	
Undergraduate college degree	78 (19.4%)	44 (22.8%)	34 (16.3%)	
Graduate degree	62 (15.4%)	31 (16.1%)	31 (14.8%)	
*Employment status*				0.03*
Not employed	144 (35.8%)	62 (32.1%)	82 (39.1%)	
Employed part-time	64 (15.9%)	25 (13%)	39 (18.7%)	
Employed full-time	194 (48.3%)	106 (54.9%)	88 (42.1%)	
*Annual household income*				0.09
Less than $5,000	55 (13.7%)	27 (14%)	28 (13.4%)	
$5,000–$19,999	60 (14.9%)	22 (11.4%)	38 (18.2%)	
$20,000–$30,999	71 (17.7%)	37 (19.2%)	34 (16.3%)	
$31,000–$50,999	89 (22.1%)	38 (19.7%)	51 (24.4%)	
$51,000–$79,999	60 (14.9%)	32 (16.6%)	28 (13.4%)	
$80,000–$100,000	23 (5.7%)	8 (3.8%)	15 (7.8%)	
More than $100,000	32 (8%)	13 (6.2%)	19 (9.8%)	
Declined to respond	12 (3%)	3 (1.6)	9 (4.3%)	
*Subjective financial situation*				0.08
“I cannot make ends meet”	106 (26.4%)	43 (22.3%)	63 (30.1%)	
“I have just enough”	210 (52.2%)	101 (52.3%)	109 (52.2%)	
“I am comfortable”	86 (21.4%)	49 (25.4%)	37 (17.7%)	
*Region of residence*				0.62
Northeast	61 (15.2%)	21 (14%)	34 (16.3%)	
Midwest	75 (18.7%)	41 (21.2%)	34 (16.3%)	
South	236 (58.7%)	111 (57.5%)	125 (59.8%)	
West	30 (7.5%)	14 (7.3%)	16 (7.7%)	
*Mother’s shares parenting responsibility*				0.48
Yes	249 (61.9%)	123 (63.7%)	126 (60.3%)	
No	153 (38.1%)	70 (36.3%)	83 (39.7%)	
*Believes co-parent would support HPV vaccination (N = 249)*				<0.001***
Yes (somewhat – strongly agree)	175 (70.3%)	113 (91.9%)	62 (49.2%)	
No (somewhat – strongly disagree)	74 (29.7%)	10 (8.1%)	64 (50.8%)	
*Number of daughters*				0.05*
1	328 (81.6%)	164 (85%)	164 (78.5%)	
2	63 (15.7%)	28 (14.5%)	35 (16.7%)	
3	8 (2%)	1 (0.5%)	7 (3.3%)	
4 or more	3 (0.7%)	0%	3 (1.4%)	
*Daughter’s age*	*M* = 11.86 (*SD* = 2.05)	*M* = 11.85 (*SD* = 1.97)	*M* = 11.87 (*SD* = 2.13)	0.96
9 to 12 years old	247 (61.4%)	120 (62.2%)	127 (60.8%)	
13 to 15 years old	155 (38.6%)	73 (37.8%)	82 (39.2%)	
*Daughter’s insurance status*				0.42
Uninsured	14 (3.5%)	9 (4.7%)	5 (2.4%)	
Public health insurance	272 (67.7%)	127 (65.8%)	145 (69.4%)	
Private health insurance	116 (28.9%)	57 (29.5%)	59 (28.2%)	
*Daughter has pre-existing health condition*				0.23
Yes	158 (39.3%)	70 (36.3%)	88 (42.1%)	
No	244 (60.7%)	123 (63.7%)	121 (57.9%)	
*Daughter’s vaccine history (out of 10 recommended vaccines)*	*M* = 5.78 (*SD* = 3.10)	*M* = 6.40 (*SD* = 2.90)	*M* = 5.21 (*SD* = 3.17)	<0.001***
*Previously heard of HPV*				0.02*
Yes	345 (85.8%)	174 (90.2%)	171 (81.8%)	
No	57 (14.2%)	19 (9.8%)	38 (18.2%)	
*Aware of HPV vaccine*				0.01**
Yes	332 (82.6%)	170 (88.1%)	162 (77.5%)	
No	70 (17.4%)	23 (11.9%)	47 (22.5%)	
*Mother’s HPV vaccine status*				<0.001***
Has received the HPV vaccine	109 (27.1%)	86 (44.6%)	23 (11%)	
Has not received HPV vaccine	253 (62.9%)	92 (47.7%)	161 (77%)	
Does not know	40 (10%)	15 (7.8%)	25 (12%)	
*Mother’s vaccine history (out of 11 recommended vaccines)*	*M* = 6.25 (*SD* = 3.10)	*M* = 6.93 (*SD* = 2.81)	*M* = 5.63 (*SD* = 3.23)	<0.001***
*Have you or anyone close to you received an abnormal pap smear result?*				0.05*
Yes	200 (49.8%)	106 (54.9%)	94 (45%)	
No or I do not know	202 (50.2%)	87 (45.1)	115 (55%)	
*Have you or anyone close to you ever had genital warts?*				0.12
Yes	67 (16.7%)	38 (19.7%)	29 (13.9%)	
No or I do not know	335 (83.3%)	155 (80.3%)	180 (86.1%)	
*Have you or anyone close to you ever developed an STI?*				0.62
Yes	145 (36.1%)	72 (37.3%)	73 (34.9%)	
No or I do not know	257 (63.9%)	121 (62.7%)	136 (65.1%)	
*Have you or anyone close to you received a cervical cancer or other HPV-related cancer diagnosis?*				0.16
Yes	80 (19.9%)	44 (22.8%)	36 (17.2%)	
No or I do not know	322 (80.1%)	149 (77.2%)	173 (82.8%)	

Statistical tests: Independent *t*-tests for participant and daughter’s age, mother’s and daughter’s vaccine history; Chi-square tests of independence for all other variables.

*p ≤ 0.05, **p ≤ 0.01, ***p ≤ 0.001.

**Table 2:** Differences in hypothesized factors of HPV vaccine intentions.

	Total sample *N* = 402	Intends to vaccinate *N* = 193 (48%)	Does not intend to vaccinate *N* = 209 (52%)	*p* value
	*M* (SD)	*M* (SD)	*M* (SD)	
*Mother’s HPV perceptions*				
HPV knowledge	9.20 (2.04)	9.44 (1.93)	8.99 (2.13)	0.03*
HPV susceptibility	3.38 (1.80)	3.72 (1.81)	2.07 (1.73)	<0.001***
HPV severity	4.76 (1.77)	5.14 (1.61)	4.40 (1.83)	<0.001***
*Mother’s vaccine attitudes*				
General vaccination attitudes	4.85 (1.43)	5.42 (1.31)	4.32 (1.33)	<0.001***
HPV vaccine benefits	4.98 (1.49)	5.70 (1.14)	4.32 (1.46)	<0.001***
HPV vaccine safety concerns	3.36 (1.49)	2.47 (1.17)	4.19 (1.26)	<0.001***
Mother’s self-efficacy	4.89 (1.14)	5.10 (1.05)	4.70 (1.20)	<0.001***
*Cues to action*				
Community norms	3.12 (1.00)	3.46 (0.91)	2.81 (0.97)	<0.001***
Mother’s peer norms	4.33 (1.15)	4.88 (1.02)	3.83 (1.02)	<0.001***
Doctor HPV vaccine recommendation	4.42 (3.18)	3.40 (5.52)	2.39 (3.55)	<0.001***
Doctor has not mentioned the HPV vaccine	227 (56.5%)	97 (50.3%)	130 (62.2%)	<0.001***
Doctor mentioned but did not recommend	83 (20.6%)	36 (18.7%)	47 (22.5%)	
Doctor mentioned and recommended	92 (22.9%)	60 (31.1%)	32 (15.3%)	
Perceived doctor’s influence	2.56 (1.02)	2.95 (0.95)	2.19 (0.95)	<0.001***
*Barriers to HPV vaccination*				
HPV vaccine inaccessibility	2.06 (0.85)	1.90 (0.87)	2.20 (0.81)	<0.001***
Cultural medical mistrust	3.57 (0.81)	3.40 (0.84)	3.73 (0.76)	<0.001***
Sexual risk and stigma concerns	2.85 (1.56)	2.57 (1.45)	3.12 (1.61)	<0.001***

Statistical tests: Independent *t*-tests for all means and chi-square tests of independence assessed percentage endorsement of doctors mentioning/recommending the HPV vaccine. HPV knowledge was scored out of a total of 13 points.

*p ≤ 0.05, **p ≤ 0.01, ***p ≤ 0.001.

**Table 3:** Bivariate associations between determinants of HPV vaccine intentions and HPV vaccine intentions.

	OR [95% CI]	*p* value
*Participant characteristics*		
Daughter’s age	1.00 [0.91, 1.10]	0.96
Mother’s age	1.01 [0.99, 1.04]	0.27
Number of daughters	0.60 [0.39, 0.92]	0.02*
Education level	1.08 [0.70, 1.66]	0.73
Employment status	1.36 [0.91, 2.06]	0.14
Income level	1.14 [1.02, 1.27]	0.02*
Financial situation	1.51 [0.96, 2.36]	0.08
Daughter’s vaccine history	1.14 [1.06, 1.22]	<0.001***
Mother’s vaccine history	1.15 [1.08, 1.23]	<0.001***
Mother’s HPV vaccine status	6.50 [3.87, 10.91]	<0.001***
Has experience with cervical cancer or other HPV-related cancer	1.42 [0.87, 2.32]	0.16
*Mother’s HPV perceptions*		
HPV knowledge	1.12 [1.01, 1.23]	0.03*
HPV susceptibility	1.23 [1.10, 1.37]	<0.001***
HPV severity	1.28 [1.14, 1.44]	<0.001***
*Mother’s vaccine attitudes*		
General vaccine attitudes	1.84 [1.56, 2.18]	<0.001***
HPV vaccine benefits	2.35 [1.92, 2.87]	<0.001***
HPV vaccine safety concerns	0.33 [0.27, 0.42]	<0.001***
Mother’s self-efficacy	1.38 [1.15, 1.66]	<0.001***
*Cues to action*		
Community norms	2.13 [1.68, 2.70]	<0.001***
Mother’s peer norms	2.99 [2.28, 3.90]	<0.001***
Doctor HPV vaccine recommendation	1.27 [1.18, 1.36]	< 0.001***
*Perceived barriers to HPV vaccination*		
HPV vaccine inaccessibility	0.65 [0.51, 0.83]	<0.001***
Cultural medical mistrust	0.60 [0.46, 0.77]	<0.001***
Sexual risk and stigma concerns	0.79 [0.69, 0.90]	<0.001***

Statistical tests: Unadjusted logistic regressions. Mother’s HPV vaccine status recoded as 0 = No and 1 = Yes. Education level recoded as 0 = High school education or less and 1 = Some college or more. Employment status recoded as 0 = Not employed and 1 = Part-or full-time employment. Financial situation recoded as 0 = Cannot make ends meet and 1 = Just enough or comfortable. OR = Odds ratio; CI = Confidence interval.

*p ≤ 0.05 **p ≤ 0.01 ***p ≤ 0.001.

**Table 4:** Multivariable associations between determinants of HPV vaccine intentions and HPV vaccine intentions.

	Step 1		Step 2		Step 3		Step 4		Step 5	
	AOR [95% CI]	*p* value	AOR [95% CI]	*p* value	AOR [95% CI]	*p* value	AOR [95% CI]	*p* value	AOR [95% CI]	*p* value
*Covariates*										
Number of daughters	0.58 [0.36, 0.94]	0.03*	0.57 [0.35, 0.93]	0.02*	0.52 [0.29, 0.93]	0.03*	0.55 [0.31, 0.99]	0.05*	0.53 [0.29, 0.98]	0.04*
Income level	1.12 [0.99, 1.26]	0.07	1.11 [0.98, 1.26]	0.09	0.99 [0.84, 1.17]	0.91	0.97 [0.82, 1.14]	0.68	0.98 [0.82, 1.16]	0.78
Daughter’s vaccine history	1.07 [0.95, 1.21]	0.24	1.06 [0.94, 1.20]	0.32	0.97 [0.84, 1.14]	0.74	0.98 [0.83, 1.15]	0.77	0.96 [0.81, 1.13]	0.62
Mother’s vaccine history	1.06 [0.94, 1.19]	0.34	1.03 [0.92, 1.17]	0.59	1.04 [0.90, 1.21]	0.60	1.03 [0.88, 1.21]	0.74	1.05 [0.89, 1.24]	0.56
Mother’s HPV vaccine status	6.23 [3.65, 10.60]	<0.001***	6.97 [4.00, 12.12]	<0.001***	6.36 [3.17, 12.77]	<0.001***	4.31 [2.10, 8.86]	<0.001***	4.08 [1.97, 8.46]	<0.001***
*Mother’s HPV perceptions*										
HPV knowledge			1.06 [0.94, 1.19]	0.38	0.94 [0.81, 1.10]	0.45	0.95 [0.81, 1.11]	0.48	0.94 [0.80, 1.11]	0.48
HPV susceptibility			1.15 [0.99, 1.33]	0.07	1.33 [1.09, 1.61]	0.01*	1.25 [1.02, 1.52]	0.03*	1.19 [0.96, 1.46]	0.11
HPV severity			1.18 [1.01, 1.37]	0.04*	1.06 [0.85, 1.31]	0.61	1.12 [0.89, 1.40]	0.34	1.11 [0.88, 1.40]	0.36
*Mother’s vaccine attitudes*										
General vaccine attitudes					1.06 [0.82, 1.37]	0.67	0.95 [0.72, 1.24]	0.69	0.99 [0.75, 1.31]	0.94
HPV vaccine benefits					1.73 [1.31, 2.30]	<0.001***	1.56 [1.15, 2.12]	0.01*	1.57 [1.16, 2.14]	0.004**
HPV vaccine safety concerns					0.36 [0.27, 0.47]	<0.001***	0.33 [0.25, 0.45]	<0.001***	0.28 [0.20, 0.40]	<0.001***
Mother’s self-efficacy					0.87 [0.67, 1.13]	0.30	0.73 [0.54, 0.99]	0.04*	0.78 [0.57, 1.06]	0.12
*Cues to action*										
Community norms							1.18 [0.83, 1.67]	0.35	1.18 [0.83, 1.68]	0.36
Mother’s peer norms							1.69 [1.15, 2.47]	0.01*	1.67 [1.15, 2.44]	0.01*
Doctor HPV vaccine recommendation							1.15 [1.04, 1.29]	01*	1.15 [1.03, 1.28]	0.02*
*Perceived barriers to HPV vaccination*										
HPV vaccine inaccessibility									1.27 [0.83, 1.97]	0.28
Cultural medical mistrust									0.97 [0.63, 1.50]	0.90
Sexual risk and stigma concerns									1.25 [0.97, 1.61]	0.08
Hosmer and Lemeshow test	𝜒2 (df) = 6.84 (8), *p* = 0.55		𝜒2 (df) = 7.66 (8), *p* = 0.47		𝜒2 (df) = 4.05 (8), *p* = 0.85		𝜒2 (df) = 4.76 (8), *p* = 0.78		𝜒2 (df) =5.21 (8), *p* = 0.74	
Nagelkerke pseudo *R*^2^	0.24		0.29		0.60		0.64		0.65	

Statistical tests: Adjusted multivariable logistic regression with five steps. Variables were entered as followed: Step 1–Covariates only, Step 2–Mother’s HPV Perceptions, Step 3–Mother’s Vaccine Attitudes, Step 4–Cues to Action, and Step 5–Perceived Barriers to Vaccination. Mother’s HPV vaccine status recoded as 0 = No or 1 = Yes. AOR = Adjusted odds ratio; CI = Confidence interval.

*p ≤ 0.05, **p ≤ 0.01, ***p ≤ 0.001.

## 3. Results

### 3.1. Demographics and participant characteristics

Table 1 provides descriptive statistics for demographics and participant characteristics reported for the total sample and by participant response to the vaccination intention outcome measure. Participants were on average 37 years old (*SD* = 7.88; 25 to 69 years old) and most attended college. A majority were employed at time of participation. However, there was considerable variability in annual household income across the sample although half of the sample subjectively rated their financial situation as “I have just enough.” Nearly 60% of participants lived in the Southern United States. At time of data collection, nearly all of the participants had previously heard of the HPV infection and were aware that there was an HPV vaccine. However, less than a third of mothers were themselves vaccinated against HPV. Further, out of a total of 11 recommended vaccines, mothers received about 6 vaccines on average (*SD* = 3.10). Vaccination uptake ranged from 36% to approximately 78% with the MMR (measles, mumps, and rubella) vaccine being most reported (*n* = 312, 77.6%). Half of the mothers also reported receiving the most recent flu vaccine (*n* = 201, 50%) and the COVID-19 vaccine (*n* = 203, 50.5%). Half of the sample also indicated experience or familiarity with abnormal pap smear results and about 60% had experience or familiarity with STIs; however, most reported no experience or familiarity with genital warts or HPV-related cancer diagnoses.

More than 80% of participants had only one daughter between the ages of 9 and 15 years old. Participants with more than one daughter in this age range reported on their oldest daughter between the ages of 9 and 15 years. On average, daughters were 11.86 years old (*SD* = 2.05). Approximately 60% of the daughters were between 9 and 12 years old and 40% were between 13 and 15 years old. The age of oldest daughters (*M* = 13.15, *SD* = 1.88) was significantly older than only daughters (*M* = 11.57, *SD* = 1.98), *t* (400) = −6.18, *p* < 0.001. Most daughters received public health insurance and about 40% had at least one preexisting health condition; most common was asthma (*n* = 96, 23.9%). Out of a total of 10 recommended pediatric vaccines, daughters received about 6 vaccines on average (*SD* = 3.10). Routine vaccination uptake ranged from 40% to approximately 79% depending on the vaccine with the varicella (chickenpox; *n* = 303, 75.4%) and MMR (measles, mumps, and rubella; *n* = 317, 78.9%) vaccines being most reported. Just over two-thirds of participants (61.9%) reported sharing parenting responsibilities with a co-parent.

### 3.2. HPV vaccination intentions among Black mothers

The sample (*N* = 402) was approximately equal with 48% (*n* = 193) of mothers intending to and 52% (*n* = 209) of mothers not intending to vaccinate their daughters. Differences in demographics and participants characteristics across HPV vaccination intentions are reported in Table 1. Mothers who intended to vaccinate their daughters were more likely to be employed full-time, believe that their co-parent would support HPV vaccination, and be less likely to have more than 1 daughter between 9 and 15 years than mothers who did not intend to vaccinate their daughters. Accepting mothers were also more likely to have previously heard of HPV, to be aware of an HPV vaccine, to have received the HPV vaccine themselves, and to know someone or have personal experience with abnormal pap smear results. These mothers also reported receiving significantly more traditionally recommended vaccines than unaccepting mothers. Likewise, daughters of mothers who were more accepting of the HPV vaccine received significantly more recommended childhood vaccines.

### 3.3. Factors associated with HPV vaccination intentions among Black mothers

Table 2 provides group comparisons across intentions to vaccinate against HPV for each of the theoretically identified factors. There were significant differences across HPV vaccination intentions on all factors in the expected directions. On average, participants scored 9.20 out of 13 or 71% correct on the HPV knowledge scale. Across the sample, knowledge scores ranged from 15.38 to 100% correct. Variability within groups was considerable as well. However, there was a significant difference in knowledge between mothers. Those who intended to vaccinate their daughter reported slightly higher HPV knowledge. These mothers also reported greater perceived HPV susceptibility and severity, more positive general pediatric vaccination attitudes, greater perceived HPV vaccine benefits, greater self-efficacy to request the vaccine, and more positive community and peer norms. More than half of the sample indicated that their daughter’s doctor has not mentioned the HPV vaccine. Relatedly, mothers who intended to get their daughter the HPV vaccine were significantly more likely to report that their daughter’s doctor recommended the vaccine and that they were more influenced by their daughter’s doctor. Mothers who did not intend to get the vaccine were more likely to report that their daughter’s doctor had not mentioned the vaccine at all. Lastly, mothers who did not intend to vaccinate their daughter reported greater HPV vaccine safety concerns and greater barriers to HPV vaccination including perceived inaccessibility, cultural medical mistrust, and sexual risk and stigma concerns. As reported in Table 3, HPV vaccine intentions were significantly associated with each independent factor in the expected directions: (1) All HPV perceptions, all vaccine attitudes, with the exception of safety concerns, and all cues to action increased the odds of favorable vaccine intentions and (2) HPV vaccine safety concerns and all perceived barriers were associated with decreased odds. Additionally, number of daughters between the ages of 9–15, daughter’s childhood vaccine history, mother’s vaccine history, and mother’s HPV vaccine status were significant covariates increasing the odds of favorable vaccination intentions.

### 3.4. Multivariable associations with HPV vaccination intentions among Black mothers

As shown in Table 4, Hosmer and Lemeshow tests indicated good fit for each step in the multivariable model assessing HPV vaccination intentions. In the final step, the number of daughters reported by participants was associated with a 47% decrease in the odds of intending to vaccinate (OR = 0.53, 95% CI [0.29, 0.98]) and mother’s HPV vaccine status was associated with 3-times greater odds of intentions (OR = 4.08, 95% CI [1.97, 8.46]). Hypothesized factors that retained independent associations in the final step were perceived HPV vaccine benefits (OR = 1.57, 95% CI [1.16, 2.14]), mother’s peer norms (OR = 1.67, 95% CI [1.15, 2.44]), doctor recommendation (OR = 1.15, 95% CI [1.03, 1.28]), and HPV vaccine safety concerns (OR = 0.28, 95% CI [0.20, 0.40]). Perceived benefits, supportive peer norms, and doctor recommendation were associated with 57, 67, and 15% increases in the odds of intending to vaccinate, respectively. By contrast, endorsing greater vaccine safety concerns was associated with a 72% decrease in the odds of favorable HPV vaccination intentions.

## 4. Discussion

In the current study, 48% of mothers intended to vaccinate their daughter while 52% did not. HPV vaccine acceptability varies in this population where endorsement ranges from 44–70% (52, 53). Intentions among Black mothers are likely comparable or lower than other racial/ethnic groups in the United States. In two diverse samples, vaccination intentions were reported among 62 and 74% of parents (32, 54). HPV vaccine acceptability varies globally. In two studies, less than a third of mothers in Japan and Hong Kong expressed intent to vaccinate their daughters (55, 56). However, our percentages are lower than the 70 and 79% of parents in Kenya and Ethiopia who reported interest in the HPV vaccine (57, 58). Overall, our findings add to a growing number of studies on vaccination intentions among Black mothers in the United States and the global literature on HPV vaccine acceptability among parents.

Among the various mother-specific and daughter-specific covariates considered in our multivariable model, mother’s HPV vaccine status and number of daughters reported by participants retained significant associations with vaccination intentions when controlling for all other factors. Mother’s HPV vaccine status, specifically, had the largest effect on HPV vaccination intentions than any other factor with a 300% increase in odds. As such, family health practitioners and women’s health care providers should actively involve mothers and other female guardians in efforts to promote HPV vaccination among this population. These efforts might include taking opportunities to provide catch-up vaccinations for eligible younger mothers and utilizing cervical cancer screenings as opportunities to promote and recommend the vaccine for adolescent daughters. On the other hand, intentions to vaccinate were lower among mothers with more than one daughter ages 9–15. This finding likely indicates difficulty navigating the HPV vaccination process for multiple eligible daughters. As such, providers should utilize evidence-based strategies to support mothers navigating this process including screening patient charts and flagging daughters eligible for the vaccine prior to health visits, administering vaccines to all eligible daughters at a single visit, and utilizing reminder/recall messages to keep the family engaged in completing the HPV vaccine series (59).

### 4.1. Mother’s HPV perceptions

*HPV knowledge* did not have as strong an association as anticipated, with over 80% of mothers indicating higher levels of knowledge about HPV and the HPV vaccine than expected based on prior research (26). Rather, our findings indicate that independent of knowledge, parents’ beliefs regarding their own child’s susceptibility to HPV and the severity of infection contribute to their intention to vaccinate their daughters against infection. Unlike previous work which suggests that both non-Black and Black parents are unaware or unconcerned about their child’s susceptibility to HPV (27, 31, 38, 39), the odds of intending to vaccinate their daughters increased by 23% in bivariate analyses among those in the current sample who perceived their daughter to be more susceptible to HPV infection and by 28% among those who believed HPV infection to have severe health consequences for their daughter. While fact sheets, waiting room videos, and conversations with health care professionals have been effective at improving general knowledge on HPV infection and vaccination among parents and knowledge-based interventions have been successful in promoting acceptability of the HPV vaccine among parents (60, 61), our findings suggest that education specific to the severity of HPV infection and susceptibility among Black girls and women are likely more relevant to current health literacy needs of this population than general HPV knowledge (62), especially among Black mothers unsure or disinclined to receive the HPV vaccination. Randomized control trials have found that cervical cancer-salient messages were associated with a change in vaccination intentions among 12% of participants with low HPV vaccine confidence (63) and providing susceptibility information instead of general HPV vaccine information was associated with greater vaccination intentions (64). Consistent with a previous study (27), however, multivariable associations for perceived severity and perceived susceptibility were not significant when attitudes toward vaccine safety and efficacy and cues to action were included in the analysis when bivariate associations were significant. Overall, the current findings are consistent with qualitative work conducted among Black parents which suggests severity of HPV infection is an important consideration for Black parents who view the HPV vaccine as a tool that can protect their daughter’s future (38).

### 4.2. Mother’s vaccine attitudes

*Mother’s Vaccine Attitudes* explained the largest percentage of variance in HPV vaccination intentions among this sample. The significant effects of HPV vaccine benefits and HPV vaccine safety are consistent with qualitative work conducted among Black parents that demonstrate the value of the HPV vaccine as a tool to protect Black young girls from severe outcomes like genital warts and cancer, despite parents’ skepticism, concern, and mistrust in response to the relative newness of the vaccine and perceived potential harm to fertility and other long-term or future health consequences (36–38, 40). In the current study, perceived vaccine benefits increased the odds of intending to vaccinate by 57% when controlling for all other factors. As such, messages specifically outlining benefits of receiving the HPV vaccine are likely useful for this population. Previous work on benefit-focused communication suggests that information about cancer prevention and HPV vaccine effectiveness are associated with increases in HPV vaccine confidence and motivation to receive the vaccine in experimental conditions (65). These messages are also particularly well-received by parents disinclined to vaccinate their children (66). Safety concerns in the current study decreased the odds of intending to vaccinate against HPV by 72% when controlling for all other factors. This is consistent with previous studies with national samples in the United States that have found health and safety concerns to be associated with a lower likelihood of vaccine intention or initiation (27–29, 31, 33). National Immunization Survey data indicate an 80% increase in HPV vaccine refusal attributed to vaccine safety concerns despite fewer reported adverse events (67). Public health messaging must combat rising safety concerns among parents in order to increase HPV vaccine confidence and willingness to vaccinate. Results of an intervention study demonstrate parental willingness to receive the HPV vaccine for children is positively impacted by exposure to HPV vaccine safety information (68). Parents in a recent study comparing attitudes between the HPV and COVID-19 vaccines, however, explain that positive media coverage for COVID-19 vaccine created more favorable attitudes for the COVID-19 vaccine while similar media content for the HPV vaccine does not seem to exist (69). Such media is needed. For Black parents, specifically, concerns about vaccine safety may also reflect overall medical mistrust stemming from the historical legacy of medical exploitation and discrimination endured by Black Americans (31, 35, 70). Public health messaging targeting this population must consider the intersecting safety and cultural medical mistrust concerns held among Black parents.

### 4.3. Cues to action

Factors associated with *Cues to Action*, including community and peer norms and physician recommendations, also had significant bivariate associations. Although community norms did not retain an independent effect in multivariate analysis, mother’s peer norms and doctor’s recommendation of the HPV vaccine did. When controlling for all other factors, the effect of peer support and doctor’s recommendation increased the odds of intending to vaccinate by 67 and 15%, respectively. These findings reflect previous qualitative work describing how Black mothers valued support of the HPV vaccine from other parents and church leaders in the community (40) and welcomed doctor recommendation when making health care decisions for their children (37–41, 70). Taken together, these results have significant implications for population-tailored public health messaging that must embrace the role of community and partnerships with trusted health care providers in Black mothers’ HPV vaccine decisions. One study of Black parents found that social networks for HPV vaccination advice were largely comprised of family members and friends (71). For this population, community forums are useful for addressing cultural concerns and mistrust (72) and also provide parents with opportunities to engage with peers supportive of pediatric HPV vaccination. Of significant concern is that among the current sample mothers reported that most doctors had either not recommended (20.6%) or mentioned (56.5%) the HPV vaccine for their daughters, suggesting that doctor reluctance to discuss the HPV vaccine early with parents is a substantial barrier to Black adolescent girls’ health that must be addressed. Consistent with our multivariable findings, previous research suggests that provider recommendations have significant influence on HPV vaccination although quality of said recommendations is largely dependent on the provider’s HPV knowledge, attitudes, and preferences (70, 73, 74). Consequently, communication training utilizing evidence-based techniques is needed to increase provider confidence to utilize announcements and other presumptive-style recommendations of HPV vaccine among this population (75–78).

### 4.4. Perceived barriers to HPV vaccination

*Perceived Barriers* were associated with vaccination intentions in unadjusted analyses but did not significantly add to the multivariable integrated model. Consistent with previous research, perceived inaccessibility of the HPV vaccine was negatively associated with HPV vaccination intentions in bivariate analyses (26, 27, 29). This may be a consequence of doctors’ failure to discuss and recommend the HPV vaccine, as described above, or difficulty navigating approval for the vaccine from their child’s health insurance providers. Consequently, public health policy must ensure that the HPV vaccine is affordable, readily available through public health coverage for children and safety-net clinics, and that population-level efforts focus on increasing parental awareness of resources for obtaining the vaccine. Cultural medical mistrust also significantly decreased the odds of intending to vaccinate against HPV in the current study, reflecting a long history of medical mistrust among Black people in the United States stemming from centuries of medical exploitation and discrimination. As such, our findings are consistent with qualitative work on Black parents who expressed lack of trust in health care providers, pharmaceutical companies, and the government and referred to historical events like the Tuskegee experiments fueling concerns that Black communities are targeted as “guinea pigs” in health research (35, 70). In experimental conditions, messaging specifically countering “distrust in the system” was significantly associated with positive attitudes toward the HPV vaccine and vaccination intent compared to control messages (79). Messages that reference specific culturally-anchored concerns would likely be useful for countering mistrust among Black parents. The current study is also consistent with previous work that parents’ concerns that the HPV vaccine would increase their daughter’s sexual risk and introduce sexual stigma is a source of hesitancy toward intending to vaccinate. Specifically among Black parents, qualitative themes have reflected concerns that vaccinating young daughters would validate notions of sexual promiscuity among Black girls which hinder acceptance of the vaccine and affect parents’ willingness to vaccinate their daughters at younger ages (35, 38, 39). Prior work among Black and non-Black parents has also found that those who do not intend to vaccinate their daughter expressed greater concerns about sexual behavior consequences with some parents even anticipating regret towards their decision if their daughter became more sexually active after receiving the HPV (26, 27, 33). Health care providers should ensure parents that there is little evidence that HPV vaccination is associated with initiation or engagement in sexual behavior (80, 81). Parents would likely benefit from messaging that specifically discusses the importance of receiving the HPV vaccine prior to engaging in sexual behavior (79). This type of tailored message has been associated with greater intentions to receive the vaccine among women compared to participants who received control messaging or messaging that focused on sexual transmission of HPV in a randomized control trial (82).

## 5. Study strengths and limitation

This study is unique in that it surveys a relatively large sample of Black mothers with unvaccinated daughters, and therefore, contributes a much-needed quantitative evaluation of Black mother’s attitudes and beliefs regarding vaccinating their young daughters against HPV infection. However, the results presented here must be interpreted within the limitations of the study. Findings are based on cross-sectional data which cannot examine longitudinal causal effects of the hypothesized predictors on intentions to vaccinate against HPV nor can the study confirm a positive relationship between plans to vaccinate and actualized vaccine uptake among this population. Further, participant recruitment and participation were conducted entirely online, and consequently, participation was limited to individuals with access to the internet on web-enabled devices and who frequently participate in online surveys for compensation. As a result, the current study may not have reached those who do not have access to the internet or are not engaged in online survey-taking. Further, demographic data suggest that while there is considerable representation across household income, a majority of the sample complete one or more years of college. Therefore, the HPV attitudes, beliefs, and vaccination intentions of those who have obtained less education may not be adequately captured in the current study. Additionally, 58.7% of the sample resided in the Southern U. S. states. However, there was no significant effect of region on HPV vaccination intentions, and further, this percentage is nationally-representative and mirrors estimates that 58.7% of the United States Black population lives in the South (83).

## 6. Conclusion

Early HPV vaccination is associated with greater vaccine efficacy and improved population-level coverage (8). However, persistent lags in vaccine uptake among parents is a concern, especially for Black girls who face increased risk of HPV infection, HPV-related outcomes, and HPV-related mortality. Additionally, sustained declines in HPV vaccination throughout the COVID-19 pandemic likely means even greater barriers to HPV vaccine initiation among Black adolescent girls. To avoid undue burden of future HPV infection and related outcomes among young Black girls, there is an urgent need to increase HPV vaccination coverage following deficits caused by the pandemic. Consequently, the implementation of evidence-based strategies such as ensuring that health care providers use all possible opportunities to recommend the HPV vaccine to girls ages 9 and older, utilization of patient reminder/recall messages to ensure initiated girls remain in care, and providing alternative access to the vaccine are recommended (84). The current study also suggests that among Black mothers, specifically, a variety of factors inform intentions to vaccinate daughters, including the mothers’ own HPV vaccine status, the number of daughters they have, perceived benefits of the HPV vaccine and perceived safety concerns, subjective peer norms surrounding HPV vaccination, and doctor’s recommendation. Therefore, these factors should be considered in efforts to increase vaccine initiation among this population. HPV knowledge was high in the sample, challenging the value of current public health campaigns that have solely focused on providing HPV infection and vaccine information. The current study suggests public messaging focused on population susceptibility and severity of infection, HPV vaccine safety concerns, and HPV vaccine efficacy may result in greater vaccine acceptance. Public health efforts may also be better focused on benefits of being vaccinated and community support. Likewise, the current findings suggest that doctors’ failure to discuss or recommend the HPV vaccine is a significant barrier to uptake among Black families, who in particular rely on their own child’s doctor to make health care decisions although they may distrust the medical establishment in general. Culturally-sensitive medical training must be a priority among health care providers of Black young girls.

## Data availability statement

The raw data supporting the conclusions of this article will be made available by the authors, without undue reservation.

## Ethics statement

The studies involving human participants were reviewed and approved by Fordham University IRB. The patients/participants provided their written informed consent to participate in this study.

## Author contributions

AG and CF: conceptualization, methodology, and funding acquisition. AG: writing-original draft, data visualization, and formal analysis. CF: supervision & mentorship and writing-review & editing. All authors contributed to the article and approved the submitted version.

## Funding

This study was supported by funding from the Center for Ethics Education at Fordham University, the American Psychological Association Division 38 Graduate Student Research in General Health Psychology Award, and the PSI CHI Mamie Phipps Clark Diversity Research Grant.

## Conflict of interest

The authors declare that this research was conducted in the absence of any commercial or financial relationships that could be construed as a potential conflict of interest.

## Publisher’s note

All claims expressed in this article are solely those of the authors and do not necessarily represent those of their affiliated organizations, or those of the publisher, the editors and the reviewers. Any product that may be evaluated in this article, or claim that may be made by its manufacturer, is not guaranteed or endorsed by the publisher.
